# Copper deficiency

**DOI:** 10.1002/jha2.401

**Published:** 2022-02-16

**Authors:** Elena E. Solomou

**Affiliations:** ^1^ Department of Internal Medicine University of Patras Medical School Rion Greece

**FIGURE 1:**
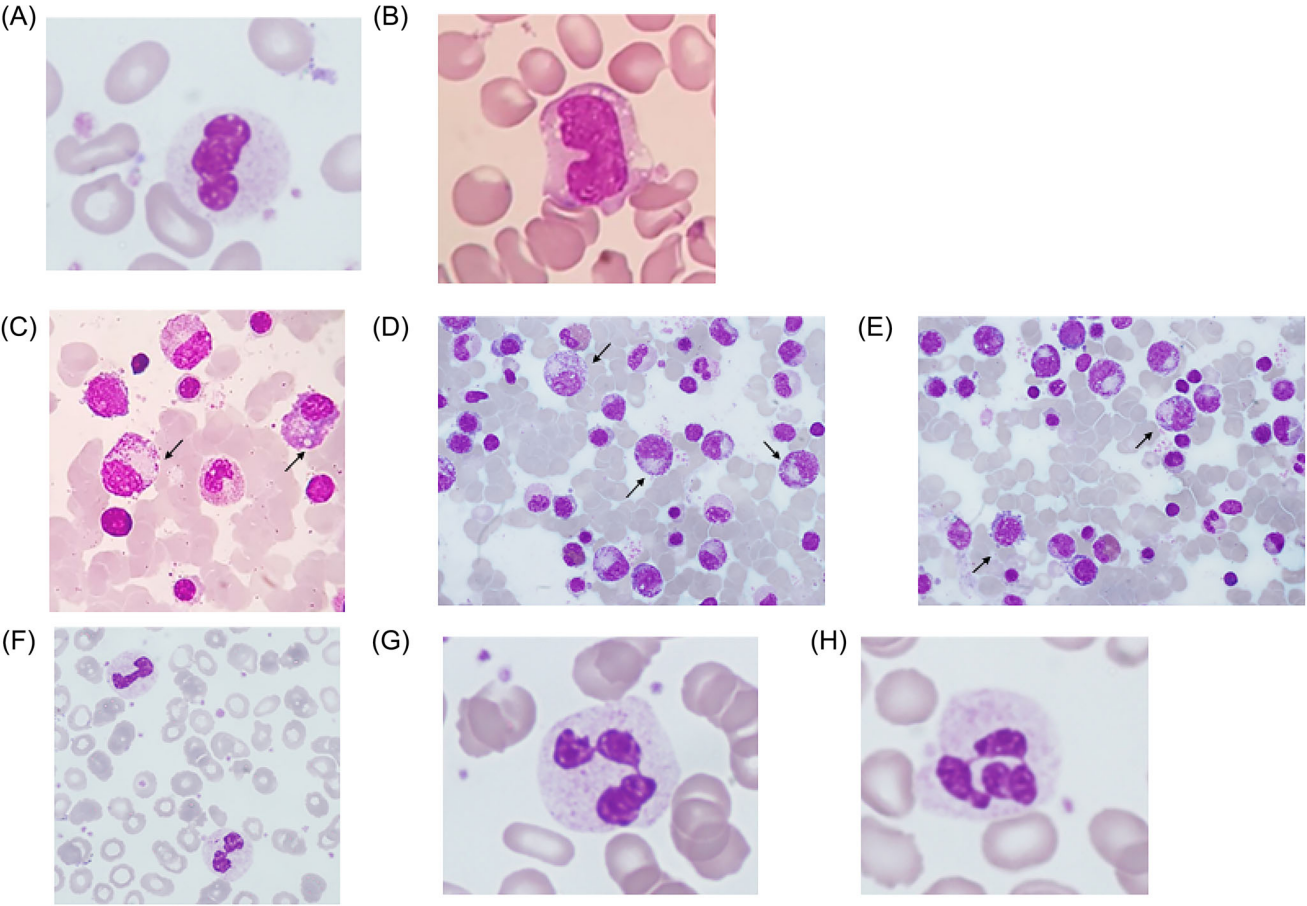
A: Abnormal nuclear globulization of neutrophils and B. cytoplasmic vacuolization of monocytes in the peripheral blood. C–E: A bone marrow smear showing asynchronous nuclear to cytoplasmic maturation, dyserythropoiesis, and abnormal distribution of granules in the cytoplasm of myeloid precursors along with cytoplasmic vacuolization (arrows). F–H: Improvement in the peripheral blood morphology two months (F), one (G) and two (H) years after initiation of treatment.

A 45 year‐old woman presented for evaluation because of neutropenia and anemia. Her complete blood counts (CBC) revealed Hb: 9 g/dl, MCV: 99 fl, WBC: 1.4 × 10^9^/L, PLT: 257 × 10^9^/L, and absolute neutrophil count: 0.45 × 10^9^/L. She was diagnosed with idiopathic autonomic neuropathy of the gastrointestinal tract since the age of 25 years. On clinical examination, the patient was afebrile, but pale. Examination of a peripheral blood smear disclosed decreased granulation along with abnormal nuclear globulization of the neutrophils (Figure 1A) and cytoplasmic vacuolization of monocytes (Figure 1B). A bone marrow smear showed asynchronous nuclear to cytoplasmic maturation, dyserythropoiesis, and abnormal distribution of granules in the cytoplasm of myeloid precursors along with cytoplasmic vacuolization (Figure 1C–E). Cytogenetics were normal. Further evaluation disclosed very low levels of ceruloplasmin and copper. Examination for Kayser–Fleisher rings was negative. A diagnosis of copper deficiency due to malabsorption was made, and the patient received oral copper supplementation resulting in a rapid improvement of CBC counts and peripheral blood morphology (Figure 1F: 2 months; G: 1 year; H: 2 years, after treatment initiation). Dysplastic features do not always represent myelodysplastic syndromes; other rare mimics, including copper deficiency, should be included in the differential diagnosis.

